# Chromosomal Targeting by the Type III-A CRISPR-Cas System Can Reshape Genomes in *Staphylococcus aureus*

**DOI:** 10.1128/mSphere.00403-17

**Published:** 2017-11-15

**Authors:** Jing Guan, Wanying Wang, Baolin Sun

**Affiliations:** a CAS Key Laboratory of Innate Immunity and Chronic Disease and School of Life Sciences and Medical Center, University of Science and Technology of China, Hefei, Anhui, China; b Hefei National Laboratory for Physical Sciences at Microscale, Hefei, Anhui, China; University of Nebraska Medical Center

**Keywords:** CRISPR-Cas system, *Staphylococcus aureus*, chromosomal targeting, mobile genetic element, staphylococcal cassette chromosome *mec*

## Abstract

CRISPR-Cas (clustered regularly interspaced short palindromic repeat [CRISPR]-CRISPR-associated protein [Cas]) systems can provide protection against invading genetic elements by using CRISPR RNAs (crRNAs) as a guide to locate and degrade the target DNA. CRISPR-Cas systems have been classified into two classes and five types according to the content of *cas* genes. Previous studies have indicated that CRISPR-Cas systems can avoid viral infection and block plasmid transfer. Here we show that chromosomal targeting by the *Staphylococcus aureus* type III-A CRISPR-Cas system can drive large-scale genome deletion and alteration within integrated staphylococcal cassette chromosome *mec* (SCC*mec*). The targeting activity of the CRISPR-Cas system is associated with the complementarity between crRNAs and protospacers, and 10- to 13-nucleotide truncations of spacers partially block CRISPR attack and more than 13-nucleotide truncation can fully abolish targeting, suggesting that a minimal length is required to license cleavage. Avoiding base pairings in the upstream region of protospacers is also necessary for CRISPR targeting. Successive trinucleotide complementarity between the 5′ tag of crRNAs and protospacers can disrupt targeting. Our findings reveal that type III-A CRISPR-Cas systems can modulate bacterial genome stability and may serve as a high-efficiency tool for deleting resistance or virulence genes in bacteria.

**IMPORTANCE** *Staphylococcus aureus* is a pathogen that can cause a wide range of infections in humans. Studies have suggested that CRISPR-Cas systems can drive the loss of integrated mobile genetic elements (MGEs) by chromosomal targeting. Here we demonstrate that CRISPR-mediated cleavage contributes to the partial deletion of integrated SCC*mec* in methicillin-resistant *S. aureus* (MRSA), which provides a strategy for the treatment of MRSA infections. The spacer within artificial CRISPR arrays should contain more than 25 nucleotides for immunity, and consecutive trinucleotide pairings between a selected target and the 5′ tag of crRNA can block targeting. These findings add to our understanding of the molecular mechanisms of the type III-A CRISPR-Cas system and provide a novel strategy for the exploitation of engineered CRISPR immunity against integrated MGEs in bacteria for clinical and industrial applications.

## Abstract (precis)

*Staphylococcus aureus* is a pathogen that can cause a wide range of infections in humans. Studies have suggested that CRISPR-Cas systems can drive the loss of integrated mobile genetic elements (MGEs) by chromosomal targeting. Here we demonstrate that CRISPR-mediated cleavage contributes to the partial deletion of integrated SCC*mec* in methicillin-resistant *S. aureus* (MRSA), which provides a strategy for the treatment of MRSA infections. The spacer within artificial CRISPR arrays should contain more than 25 nucleotides for immunity, and consecutive trinucleotide pairings between a selected target and the 5′ tag of crRNA can block targeting. These findings add to our understanding of the molecular mechanisms of the type III-A CRISPR-Cas system and provide a novel strategy for the exploitation of engineered CRISPR immunity against integrated MGEs in bacteria for clinical and industrial applications.

## INTRODUCTION

*Staphylococcus aureus* is a bacterial pathogen that can cause infectious diseases in humans, ranging from skin or soft tissue infections to life-threatening illnesses (1). Recent studies have revealed that the emergence and resurgence of methicillin-resistant *S. aureus* (MRSA) are serious public health threats, especially the community-associated MRSA infections (2). Mechanisms of resistance to β-lactam antibiotics among MRSA strains are due to the acquisition of the *mecA* resistance gene, which is carried on staphylococcal cassette chromosome *mec* (SCC*mec*) and encodes an additional penicillin-binding protein PBP2a with low affinity for β-lactam antibiotics (3–5). The mobile genetic element SCC*mec* can conduct horizontal transfer among staphylococcal strains and accordingly lead to the prevalence of methicillin resistance (6).

Clustered regularly interspaced short palindromic repeats (CRISPRs) and CRISPR-associated proteins (Cas) constitute an adaptive immunity system that protects archaea and bacteria from threats of foreign mobile elements. According to the constitution and function of Cas proteins, CRISPR-Cas systems are currently classified into five distinctive types and diverse subtypes (7). Studies have mainly focused on types I, II, and III systems in the last decade. CRISPR loci, composed of conserved repeats and diverse spacers, are under the control of an AT-rich leader sequence. Repeats and spacers are first transcribed into precursor CRISPR RNAs (pre-crRNAs) and then are processed into small and mature crRNAs, which can guide the Cas complex for sequence-specific targeting (8–10). A recent study revealed that pre-crRNA processing is independent on its sequence, length, or secondary structure in *Staphylococcus epidermidis* type III-A CRISPR-Cas system (11). The protospacer adjacent motif (PAM) and seed sequence play a key role in recognition and targeting (12, 13), as well as new spacer acquisition (14) in type I and type II CRISPR-Cas systems. Type III systems do not require a PAM, and self/nonself discrimination relies on eight nucleotides of repeat sequence present at the 5′ handle of crRNA (crRNA 5′ tag). One early study has concluded that the 5′-tag noncomplementarity of protospacers and crRNAs at specific positions is responsible for interference, whereas extended pairing between the 5′ tag of crRNA and the target prevents autoimmunity in *S. epidermidis* (15). Similar results were observed but at different pairing positions in *Sulfolobus solfataricus* (16). However, until now, the role of a potential seed sequence for type III immunity has remained unknown. Intriguingly, a previous study implies that exact complementarity between crRNAs and protospacers in the 5′ end is necessary for antiplasmid immunity in *S. aureus* type III-A system (17).

Most of the spacers from multiple organisms are characterized to be homologous to the sequences derived from bacteriophages or conjugative plasmids, but a number of spacers are also found to match with archaeal or bacterial genomes. It has been reported that 59 of 330 CRISPR-positive organisms possess at least one spacer targeting endogenous genomic sequence (18), indicating that incorporation of a self-targeting spacer is not an accident. Another study suggests that among 4,500 spacers from various organisms, 35% have homologs to chromosomal sequences in the NCBI database (19). Some of these spacers target genes within integrated mobile genetic elements (MGEs), while others target nonmobile genes. For example, *Pectobacterium atrosepticum* contains a self-targeting spacer completely complementary to an endogenous gene within a horizontally acquired island named HAI2 (20). It has also been found that a spacer matches the sequence within *hisS*, which codes for the histidyl-tRNA synthetase in *Pelobacter carbinolicus* (21). These findings raise the question of what role self-targeting spacers may play. One controversial idea is that self-targeting spacers may participate in gene regulation and bacterial genome evolution (22, 23). A few authors proposed that chromosomal targeting has a deleterious effect, but bacteria can survive at the cost of the disruption of CRISPR arrays or Cas proteins (20, 21, 24). They were disposed to agree with the view that chromosomal targeting is a case of autoimmunity rather than a regulatory mechanism (18). Although incorporation of a self-targeting spacer is less common than spacers against MGEs, this phenomenon provides an insight into the biological application of CRISPR-Cas systems.

The interaction between the CRISPR-Cas system and prophage has been a subject of intense research in the last 10 years. Marraffini et al. pointed out numerous novel views about antibacteriophage immunity in *S. epidermidis* type III-A CRISPR-Cas system (25–27). Unfortunately, an active prophage in the CRISPR-positive *S. aureus* has not been found yet. A recent study concluded that CRISPR-negative strains contained signiﬁcantly more prophages and larger genomes than the CRISPR-positive strains did (28). A possible reason is that the uptake of MGEs is prevented by CRISPR-Cas systems. A few studies actually supported this hypothesis. As the consequence of transforming an engineered plasmid with spacers targeting the chromosomal gene within HAI2, *P. atrosepticum* survived by excision of the entire HAI2 island or deletion of part of the pathogenicity island (20). A similar result has been observed in the *Streptococcus thermophilus* that carries a type II-A CRISPR-Cas system (29). When a plasmid with spacers targeting genomic islands was transformed, CRISPR-Cas systems can drive deletion of large genomic islands and genome evolution by insertion sequence (IS)-dependent recombination. Collectively, these observations indicate that CRISPR-Cas systems can direct bacterial genome rearrangement and evolution through deletion of the integrated MGEs. Spontaneous SCC*mec* excision events occur at a low frequency in the wild-type population (30, 31).

CRISPR-Cas systems have been found in several *S. epidermidis* and *S. aureus* strains, especially in SCC*mec*-positive strains (32–35). It has been demonstrated that CRISPR-Cas systems can limit plasmid conjugation and phage invasion in *S. epidermidis* strain RP62A (26, 36). In a previous study, we identified six clinical isolates of *S. aureus* that harbor type III-A CRISPR-Cas systems and demonstrated their immunity function (17). Here, we further performed experiments in *S. aureus* strain AH1, a methicillin-resistant clinical isolate containing type V SCC*mec*. To investigate the effect of CRISPR-mediated chromosomal targeting toward SCC*mec*, we constructed artificial CRISPR plasmids with spacers targeting the *mecA* gene within SCC*mec*. Our results demonstrate that spacers with a perfect match to the endogenous gene are actually detrimental, but bacteria can avoid this autoimmunity by various mutations. The most common mutation mechanism was reshaping the sequence within SCC*mec* instead of driving excision of the entire SCC*mec*. We further found that the appropriate length of crRNAs and successive mismatches between the 5′ tag of crRNAs and nucleotides adjacent to protospacers are required for type III-A CRISPR immunity. These findings provide novel insight into the molecular mechanisms of CRISPR targeting and clinical applications of CRISPR-Cas systems in the treatment of MRSA infection.

## RESULTS

### Determination of the functional CRISPR promoter region.

**FIG 1:**
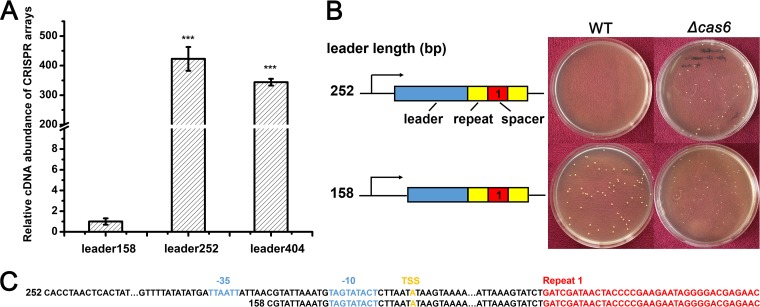
Identification of the functional CRISPR promoter region. (A) Relative transcription level of the native CRISPR array under the control of the truncated leader in the CRISPR knockout strain. The lengths of truncated leader were 404, 252, and 158 bp. Values that are significantly different from the value for the leader158 (*P* < 0.001) are indicated by three asterisks. (B) Artificial mini-CRISPR arrays with truncated leaders of 158 and 252 bp were constructed and transformed into the WT and *cas6* knockout strains. At least three independent transformation experiments were performed, and representative plates are shown. (C) The sequences of the truncated 252-bp and 158-bp leaders, the predicted −35 and −10 promoter regions (blue), and the transcription start site (TSS) (in orange) relative to the first CRISPR repeat (red) are shown.

**FIG 2:**
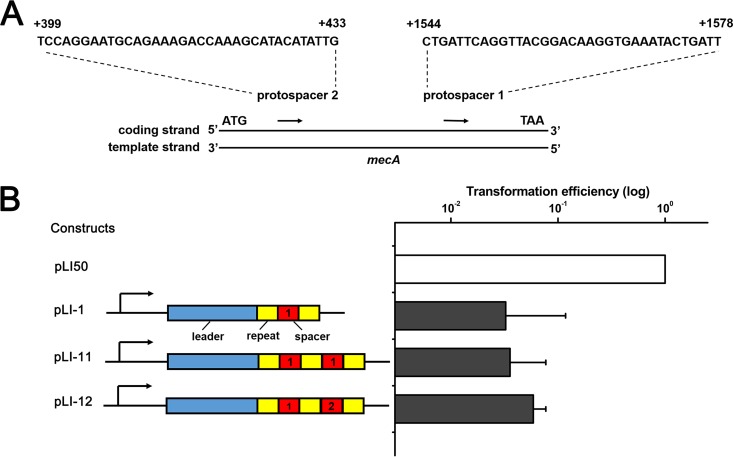
One chromosome-targeting spacer is sufficient for CRISPR targeting. (A) Schematic of two sequence regions selected as an artificial CRISPR array-targeting site. Sequences of the coding strand from 1544 to 1578 nt and from 399 to 433 nt relative to the start codon (ATG) of *mecA* constituted protospacer 1 and protospacer 2, respectively. (B) *mecA*-targeting constructs pLI-1 (one spacer), pLI-11 (two identical spacers), and pLI-12 (two different spacers) displayed similar toxicity. The transformation efficiency of the empty plasmid pLI50 (no spacer) was set at 100%. Transformations were performed three times, and average relative transformation efficiencies plus standard deviations (error bars) are shown in the graph.

To investigate the effect of chromosomal targeting by the type III-A CRISPR-Cas system in *S. aureus* strain AH1, we constructed artificial CRISPR plasmids containing chromosome-targeting spacers. We first identified the functional promoter region of the CRISPR array by constructing a series of plasmids with truncated leader sequences of 404, 252, and 158 bp of the first repeat and native CRISPR arrays. These plasmids were transformed into the CRISPR knockout strain, and the transcription efficiencies of different leader sequences were detected by real-time quantitative reverse transcription-PCR (qRT-PCR). The transcriptional level of native crRNAs driven by the 158-bp leader sequence decreased more than 300-fold (Fig. 1A). Then, we constructed artificial CRISPR plasmids with 252-bp or 158-bp leader sequence and a mini-CRISPR array generating crRNAs targeting *mecA*, yielding plasmids pLI-252 and pLI-158. The *mecA* gene is located on SCC*mec* and encodes an alternative penicillin-binding protein PBP2a, which exhibits a much lower affinity to β-lactam antibiotics than PBP2 does (4). These two plasmids were transformed into the wild-type (WT) and *cas6* knockout strains. Transformation results showed that only the 252-bp leader sequence exhibited obvious transcriptional activity, which was detrimental to bacterial cell growth (Fig. 1B). The low transcription efficiency of the 158-bp leader may be due to its position that is too close to the predicted −35 and −10 promoter regions and the putative transcription start site of the CRISPR array (Fig. 1C). As a result, the 252-bp leader was chosen as the promoter of the artificial CRISPR array in our research. The targeting activity of artificial CRISPR plasmids was assessed by the transformation efficiency relative to the transformation efficiency of the empty plasmid pLI50. There was no apparent additional effect with the *mecA*-targeting constructs containing one spacer (pLI-1), two identical spacers (pLI-11), or two individual spacers (pLI-12) (Fig. 2), suggesting that a single spacer is sufficient for targeting.

### Chromosomal targeting by the type III-A CRISPR-Cas system in *S. aureus*.

**FIG 3:**
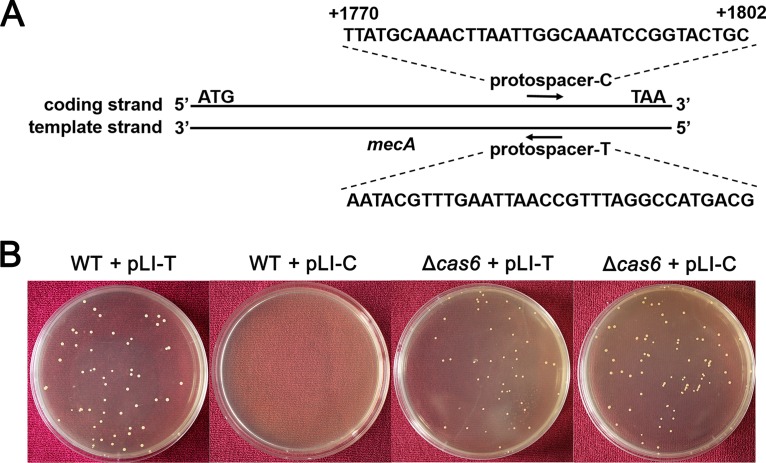
An artificial CRISPR plasmid with spacers targeting *mecA* displays Cas-dependent toxicity. (A) Schematic of sequence regions selected as artificial CRISPR plasmid targeting sites. The sequence of protospacer-C is in the coding strand of *mecA*, whereas protospacer-T is the complementary sequence of protospacer-C in the template strand. (B) Transformation plates of the WT strain and the Δ*cas6* mutant strain after growth for 36 h on TSB containing chloromycetin (Chl). CRISPR plasmids contained spacers targeting the coding strand and template strand of *mecA*.

To further investigate whether the effect of chromosomal targeting by the type III-A CRISPR-Cas system is dependent on the transcription of the target gene, we constructed artificial CRISPR plasmids with spacers targeting the coding strand (pLI-C) and the template strand (pLI-T) of *mecA* and transformed them into the WT and *cas6* knockout strains (Fig. 3). The results indicated that nearly no transformant was obtained in the WT strain with spacers targeting the coding strand of *mecA*, whereas many transformants were obtained with spacers targeting the template strand of *mecA*, and many transformants were obtained when CRISPR immunity was abolished in the *cas6* knockout strain (Fig. 3B) (17). In addition, we detected the oxacillin MIC level of transformants generated from the *cas6* knockout strain. The transformants exhibited the same MIC level with the WT and *cas6* knockout strains (Table 1), indicating again that Cas6 is essential for immunity function. These results demonstrate that chromosomal targeting by the type III-A CRISPR-Cas system is dependent on the transcription of the target gene.

**TABLE 1:** Oxacillin susceptibility of *S. aureus* strains

Strain and relevant characteristic(s)	Oxacillin MIC (mg/liter)^a^
AH1 strains	
WT	2
Containing CRISPR plasmid; *mecA* deletion	<0.5
Containing CRISPR plasmid; *cas* mutation	2
Containing destroyed CRISPR plasmid	2
Δ*cas6* strains	
*S. aureus* AH1; *cas6*-deleted strain	2
*S. aureus* AH1, *cas6*-deleted strain; containing CRISPR plasmid	2

a Oxacillin MIC in Mueller-Hinton broth.

**FIG 4:**
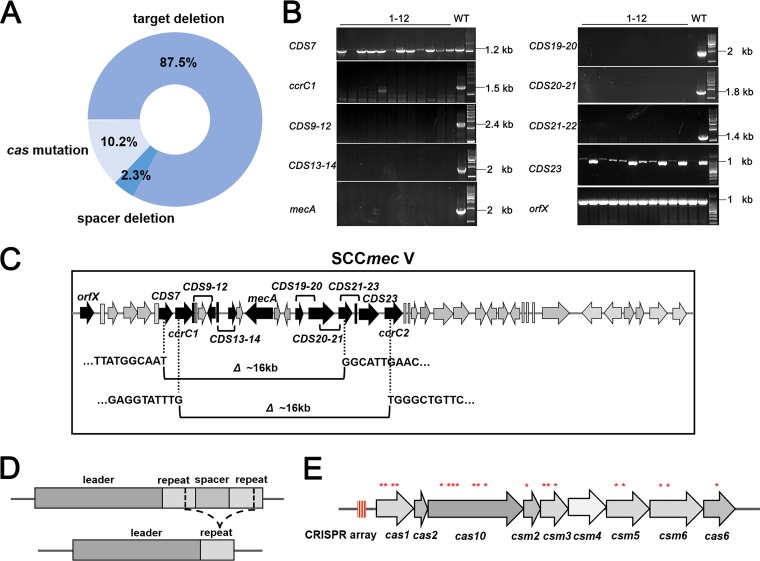
Transformants evade CRISPR targeting by different mutations. (A) Summary of different mutation types and corresponding proportions of 128 surviving clones. (B) PCR amplification for identification of large fragment deletions across SCC*mec*. Deletions occurred between *CDS7* and *CDS23*. The weaker PCR bands reflected gene breaking regions. 1-12, lanes 1 to 12. (C) Schematic of two representative transformants contained about 16-kb deletion within SCC*mec*. The deletion regions were between *CDS7* and *CDS21* and between *ccrC1* and *ccrC2*. (D) Schematic of transformants avoiding CRISPR attack by removal of the spacer repeat unit. (E) Distribution of mutations within different *cas* genes. Red asterisks indicate the mutation sites of single-nucleotide insertions, deletions, or substitutions.

The chromosome-targeting spacers displayed extremely high chromosomal targeting capacity, leading to the death of more than 95% of the transformed bacterial cells. The surviving clones evaded CRISPR attack by various mutations. To distinguish the mutations, we analyzed 128 transformants that had been obtained in several transformation experiments. Mutation analysis was implemented by determining the presence of any mutation in the target, CRISPR plasmid, or *cas* genes. We extracted genomic DNA from all transformants and amplified *mecA* as well as its surrounding regions by PCR. Surprisingly, large fragment deletions of similar sizes across the targeted region occurred in more than 87% of the transformants (Fig. 4A and B). To map the accurate deletion region, we randomly chose two transformants to perform whole-genome sequencing, and reads were mapped to the reference genome sequence using software. Sequence analysis revealed the deletion of fragments (~16 kb) within SCC*mec* (Fig. 4C). The deleted fragments contain 15 to 17 coding sequences (CDS) and constitute ~0.55% of the 2,900-kb genome of *S. aureus*.

We further sequenced the *mecA* PCR products from the transformants harboring *mecA* and found that no nucleotide mutation occurred in the matching region. The remaining transformants survived due to the deletion of the anti-*mecA* spacers or mutations in *cas* genes required for targeting. We found three transformants with deletion of anti-*mecA* spacer repeat unit within the impaired CRISPR constructs, which presumably occurred via recombination of repeat sequences (Fig. 4A and D). To assay inactivating mutations, we amplified the full CRISPR-Cas loci of the remaining 13 transformants and found 10 amplicons containing mutations (Fig. 4A). Sequencing results of the PCR products identified the loss-of-function mutations in different *cas* genes, including *cas1*, *cas10*, *csm2*, *csm3*, *csm5*, *csm6*, and *cas6* (Fig. 4E and Table 2). Intriguingly, we obtained three transformants with the chromosome-targeting spacer and corresponding protospacer, but no mutation was observed in *mecA*, the CRISPR array, or *cas* genes. In addition, we detected the oxacillin MIC level of all transformants. Transformants in which *mecA* was deleted were all sensitive to oxacillin, and transformants with mutations in *cas* genes or CRISPR plasmids were still resistant to oxacillin and displayed the same MIC level as the WT strain (Table 1).

**TABLE 2:** Characteristics of *cas* mutations in *S. aureus* AH1 transformants

Mutation site	Mutation type(s)	No. of transformants
*cas1*	Nucleotide substitution	3
	Nucleotide insertion, frameshift	1
*cas6*	Nucleotide insertion, frameshift	1
*cas10*	Nucleotide substitution	1
	Nucleotide insertion, frameshift	2
	Nucleotide deletion, frameshift	1
*csm2*	Nucleotide substitution	1
*csm3*	Nucleotide substitution	2
	Nucleotide insertion, frameshift	1
*csm5*	Nucleotide insertion, frameshift	1
	Nucleotide deletion, frameshift	1
*csm6*	Nucleotide insertion, frameshift	1
	Nucleotide deletion, frameshift	1

### The lengths of mature crRNAs were constant.

**FIG 5:**
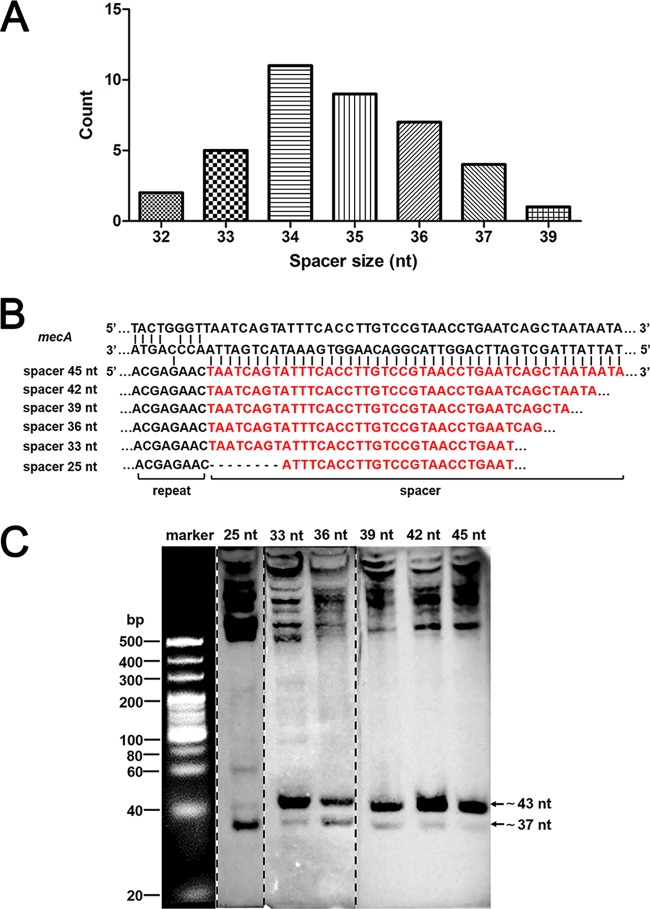
Determination of the intermediate products and mature crRNAs generated from *mecA*-targeting CRISPR arrays by Northern blotting. (A) Size distribution of 39 spacers from six CRISPR-positive *S. aureus* strains. (B) Schematic of sequences with *mecA*-targeting spacers of different lengths (red). (C) The processed crRNAs generated from spacers of different lengths showed similar sizes. The arrows indicate the positions of mature crRNAs with sizes of ~37 nt and ~43 nt. The higher bands indicate the intermediate products. Portions of the gel were taken from three different gels and joined together. The broken lines show the spliced portions.

*S. aureus* strain AH1 harbors three distinct spacers, one of which was 35  nucleotides (nt) long and two were 37 nt long (17). Characterization and comparison of 39 spacers from six CRISPR-positive *S. aureus* strains (AH1, AH2, AH3, SH1, SH2, and SH3) indicate that the size of the spacer was not constant, with the longest spacer being 39 nt, the shortest spacer being 32 nt, and the most common sizes being 34 and 35 nt (Fig. 5A). The range of spacer size was variable among different species. The longer spacers were observed in *Methanopyrus kandleri*, which possesses 51- to 72-nt spacers. In some bacteria, the spacer size is even less than 30 nt (37). To determine whether spacer size can affect crRNA processing, we introduced a series of *mecA*-targeting CRISPR arrays with spacers of different lengths and distinguished the lengths of crRNAs by Northern blotting. We found that the transcripts of artificial CRISPR arrays of different sizes were all processed into two mature crRNAs that were comparable in size (Fig. 5B and C). These results indicate that the plasmid-borne CRISPR array can be successfully transcribed and processed into mature crRNAs and that the size of the spacer is not the critical factor in crRNA processing. More interestingly, the primary CRISPR transcript with a spacer length of less than 30 nt showed a stronger hybridization signal than the 37-nt band did (Fig. 5C).

To precisely determine the sizes and sequences of mature crRNAs, we performed 5′ and 3′ rapid amplification of cDNA ends (RACE). Our RACE data indicated that all primary CRISPR transcripts were reduced to mature crRNAs with sizes of 43 and 37 nt (Table 3). The sequence of the first 8 nt (ACGAGAAC) of mature crRNAs was constant, and this crRNA 5′ tag was conservative in staphylococci (11). The 3′ end of crRNAs differed and maturation followed a rule that primary CRISPR transcripts were trimmed on the 3′ end and retained the 35 or 29 nt following the 5′ tag *in vivo* (Table 3). These data suggested that maturation of crRNAs is independent of the sequence and length of intermediate crRNAs and that the crRNA 3′ end maintained a constant distance from its 5′ tag (11).

**TABLE 3:** Sequences and sizes of mature crRNAs with different length spacers

CRISPR plasmid spacer length (nt)	Mature crRNA sequence^a^	crRNA size (nt)
36	ACGAGAACUAATCAGUAUUUCACCUUGUCCGUAACCUGAAUCA	43
	ACGAGAACUAATCAGUAUUUCACCUUGUCCGUAACCU	37
39	ACGAGAACUAATCAGUAUUUCACCUUGUCCGUAACCUGAAUCA	43
	ACGAGAACUAATCAGUAUUUCACCUUGUCCGUAACCU	37
42	ACGAGAACUAATCAGUAUUUCACCUUGUCCGUAACCUGAAUCA	43
	ACGAGAACUAATCAGUAUUUCACCUUGUCCGUAACCU	37
45	ACGAGAACUAATCAGUAUUUCACCUUGUCCGUAACCUGAAUCA	43
	ACGAGAACUAATCAGUAUUUCACCUUGUCCGUAACCU	37
33	ACGAGAACUAATCAGUAUUUCACCUUGUCCGUAACCUGAAUGA	43
	ACGAGAACUAATCAGUAUUUCACCUUGUCCGUAACCU	37
25	ACGAGAACAUUUCACCUUGUCCGUAACCUGAAUGAUCGAUAAC	43
	ACGAGAACAUUUCACCUUGUCCGUAACCUGAAUGAUC	37
23	ACGAGAACAUUUCACCUUGUCCGUAACCUGAGAUCGAUAACUA	43
	ACGAGAACAUUUCACCUUGUCCGUAACCUGAGAUCGA	37
22	ACGAGAACAUUUCACCUUGUCCGUAACCUGGAUCGAUAACUAC	43
	ACGAGAACAUUUCACCUUGUCCGUAACCUGGAUCGAU	37
21	ACGAGAACAUUUCACCUUGUCCGUAACCUGAUCGAUAACUACC	43
	ACGAGAACAUUUCACCUUGUCCGUAACCUGAUCGAUA	37
20	ACGAGAACAUUUCACCUUGUCCGUAACCGAUCGAUAACUACCC	43
	ACGAGAACAUUUCACCUUGUCCGUAACCGAUCGAUAA	37
17	ACGAGAACAUUUCACCUUGUCCGUAGAUCGAUAACUACCCCGA	43
	ACGAGAACAUUUCACCUUGUCCGUAGAUCGAUAACUA	37

a Spacer sequences are underlined.

### Spacer size played an important role in CRISPR targeting.

**FIG 6:**
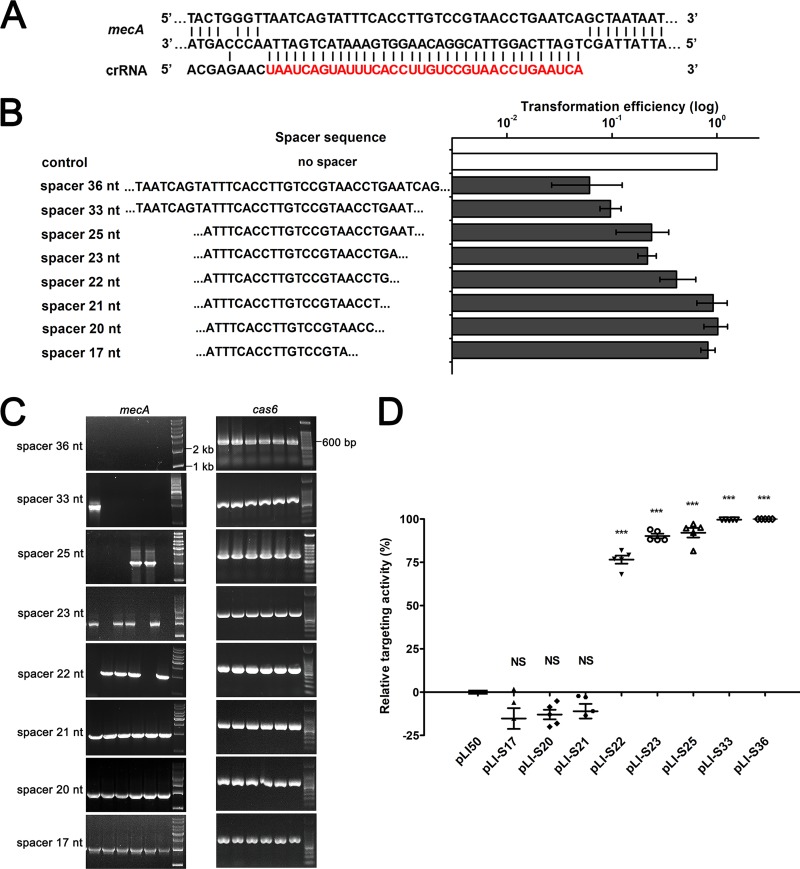
The spacer length has influence on targeting activity. (A) Schematic of base pairing between mature crRNA generated from pLI-S36 and its target sequence. Nucleotides from the spacer are highlighted in red. (B) The relative transformation efficiencies of *S. aureus* strain AH1 with artificial CRISPR plasmids containing spacers of different lengths. Transformations were performed at least three times. The transformation efficiency of the control (pLI50) was set at 100%. (C) PCR amplification for the detection of the *mecA* target gene. PCR performed for *cas6* was shown as a control. (D) Relative activity of *mecA*-targeting constructs containing spacers of different lengths. The targeting activity of empty plasmid pLI50 was set at zero. Five independent transformants were analyzed for each construct. The values are means ± standard deviations (error bars). Values that are significantly different (*P* < 0.001) from the value for pLI50 are indicated by three asterisks. Values that are not significantly different (NS) from the value for pLI50 are also indicated.

While the variation in spacer size had no influence on crRNA processing, the mature crRNAs had multiple mismatches with the protospacer sequence of *mecA*, especially when the length of the spacer was reduced (Fig. 6A and B). To investigate whether these mismatches abolish CRISPR-mediated immunity, we performed transformation experiments and detected the transformation efficiencies of each CRISPR construct (17). The results indicated that CRISPR plasmids with 33-nt (pLI-S33) and 36-nt spacers (pLI-S36) exhibited obvious targeting capacity. The relative transformation efficiencies of pLI-S36 and pLI-S33 were only about 5% (Fig. 6B). CRISPR plasmids with spacers ranging in size from 22 to 25 nt (pLI-S22, pLI-S23, and pLI-S25) displayed strong reductions in targeting capacity. The relative transformation efficiencies of pLI-S22, pLI-S23, and pLI-S25 were about 20% to 40% (Fig. 6B). CRISPR plasmids with spacer lengths of less than 21 nt had no effect on targeting. The transformation efficiencies of pLI-S17, pLI-S20, and pLI-S21 were comparable to that of the control pLI50 (Fig. 6B). To further determine the targeting capacity of these CRISPR plasmids, the presence of *mecA* for each transformant was detected by PCR ampliﬁcation, and *cas6* was amplified as a control (Fig. 6C). Unexpectedly, crRNAs and *mecA* coexisted in the daughter clones of the transformants containing *mecA*-targeting construct pLI-S17, pLI-S20, or pLI-S21, suggesting that the truncation of spacers may cause the loss of targeting activity (Fig. 6C). In the daughter clones of the transformants containing pLI-S22, pLI-S23, or pLI-S25, some lost the target gene *mecA*, while others did not (Fig. 6C). In contrast, *mecA* was deleted in all the daughter clones of the transformants containing pLI-S33 and pLI-S36 (Fig. 6C). We assumed that CRISPR targeting was partially impaired due to the truncation of spacers. Therefore, we detected the positive ratio of *mecA* in each transformant population, and the result was consistent with our hypothesis. CRISPR plasmids with spacer lengths of less than 21 nt (pLI-S17, pLI-S20, and pLI-S21) showed no targeting activity, and the transformant populations were all *mecA*-positive clones (Fig. 6D). CRISPR plasmids with 22-nt, 23-nt, and 25-nt spacers displayed higher targeting activities. The average targeting activities of pLI-S22, pLI-S22, and pLI-S25 were about 75%, 85%, and 90%, respectively (Fig. 6D). CRISPR plasmids with 33-nt and 36-nt spacers exhibited strong targeting activities. The average targeting activities of pLI-S33 and pLI-S36 were more than 99% (Fig. 6D). Altogether, these data suggest that appropriate spacer size is required for CRISPR targeting and that targeting capacity is positively associated with the spacer length within a certain range.

### Mutations in the 5′ tag of crRNAs can partially block CRISPR targeting.

In *S. epidermidis*, CRISPR immunity against nonself targets is enabled by mismatches between the 5′ upstream sequence of target DNA and crRNAs. Formation of at least three base pairings at positions −4, −3, and −2 eliminates targeting. Self-recognition and protection are achieved by complementarity between the CRISPR locus and the crRNAs. Disruption of base pairings at positions −4 and −3 or −3 and −2 abolishes protection (15).

**FIG 7:**
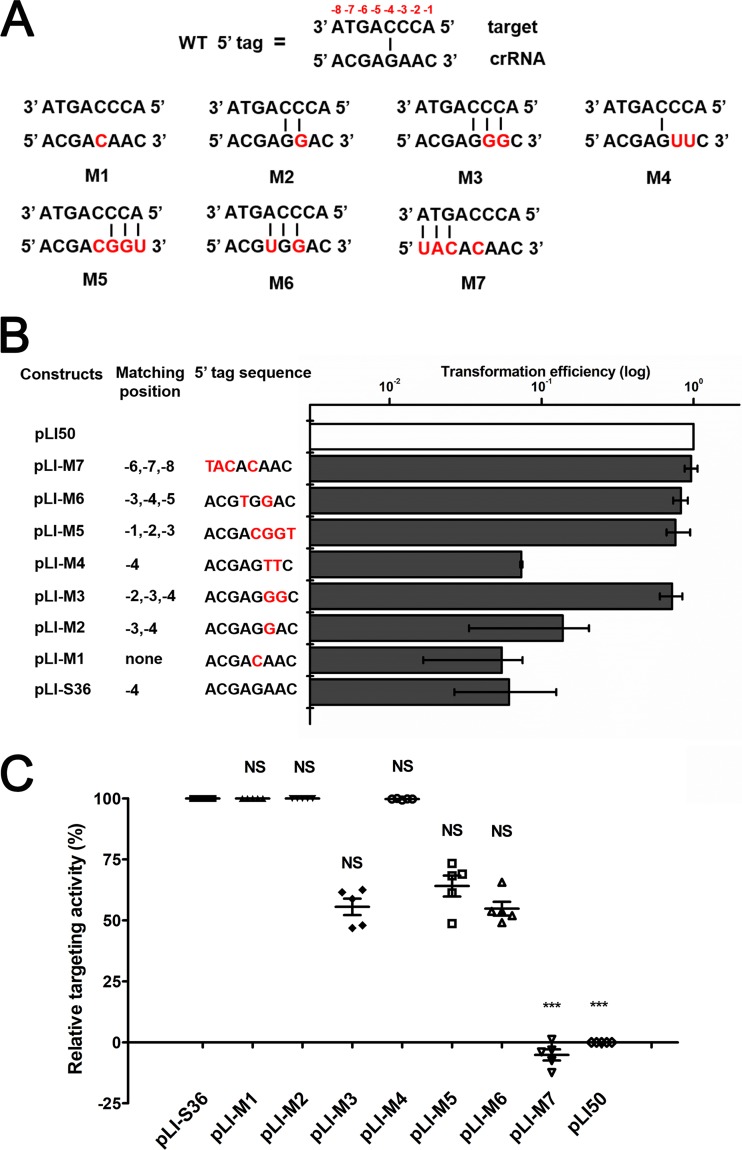
Mutations in the crRNA 5′ tag eliminate CRISPR attack. (A) Schematic of the complementarity between the flanking sequences (positions −1 to −8) of crRNAs (bottom) and target DNA (top). The mutated nucleotides are shown in red. (B) Effects of progressive sequence mutations in the 5′-tag sequence on transformation efficiency. The transformation efficiency of the empty plasmid pLI50 was set at 100%. The construct pLI-S36, which contains the native repeat, was used as a positive control. The mutated nucleotides are shown in red. (C) The relative targeting activity of *mecA*-targeting constructs contained a series of mutations in the 5′-tag sequence. The targeting activity of empty plasmid pLI50 was set at zero. The construct pLI-S36 was taken as a positive control. Five independent transformants were analyzed for each construct with bars indicating standard deviations.

To further verify this hypothesis in the *S. aureus* type III-A CRISPR-Cas system, some mutations were introduced into the upstream repeat sequence of pLI-S36, yielding a variety of complementary sequences between the crRNAs and associated protospacers (Fig. 7A). The 5′ tag of crRNAs generated from the *mecA*-targeting CRISPR construct pLI-S36 exhibited pairing with protospacers at position −4, but it did not influence CRISPR targeting (Fig. 7B). It was possible that a single nucleotide mutation was not sufficient to completely block CRISPR targeting. We then introduced some mutations at positions −2 to −4 within the 5′ tag of crRNAs. The transformation results indicated that noncomplementarity (G-4C) between the crRNA and the upstream flanking sequences of protospacer can absolutely ensure targeting (Fig. 7B). Base pairings at positions −4 and −3 (A-3G) did not significantly disrupt CRISPR targeting, whereas three consecutive matches at positions −2 to −4 (A-2G and A-3G) almost eliminated targeting (Fig. 7B). To confirm that the decisive requirement for targeting is noncomplementarity with the crRNA 5′ tag rather than nucleotide identity, we introduced mutations at the same positions (−2 and −3) but with nucleotide T, not G, and the result was consistent with our hypothesis. In contrast to mutation M3 (A-2G and A-3G), mutation M4 (A-2T and A-3T) yielded base pairing only at position −4 and could not eliminate CRISPR targeting (Fig. 7B).

To identify the positions important for protection, we introduced three consecutive nucleotide pairings at different positions in the 5′ tag (M5, M6, and M7) (Fig. 7A). Contrary to a previous report (15), mutations M5 and M6 exhibited the same transformation efficiencies as the mutation M3 did, suggesting that any three consecutive matches at positions −1 to −5 could protect the target from degradation (Fig. 7B). Mutation M7 showed nearly the same transformation efficiency as the negative control did (Fig. 7B). It was possible that mutations at positions −6, −7, and −8 eliminated crRNA maturation and targeting (15). To investigate how spacer sequence may affect CRISPR attack in the presence of a 5′-tag mutation, we detected the relative targeting activity of crRNAs with mutations in the 5′ tag. The crRNAs generated from constructs pLI-M1, pLI-M2, and pLI-M4 showed the similar targeting capacities as pLI-S36 did (Fig. 7C), which could fully degrade protospacers. In contrast, crRNAs generated from constructs pLI-M3, pLI-M5, and pLI-M6 exhibited significantly reduced targeting activities, and only ~40% to 50% protospacers were cleaved (Fig. 7C), revealing that at least three consecutive matches at positions −1 to −5 could partially disturb CRISPR targeting and protect protospacers from degradation. As expected, the pLI-M7 construct displayed no targeting activity, as did the empty vector pLI50 (Fig. 7C).

Taken together, these results demonstrate that the 5′-tag sequence can play an important role in the recognition of self/nonself. In addition, three consecutive base pairings between the 5′ tag of crRNAs and protospacer-adjacent sequences have a negative effect on CRISPR targeting.

## DISCUSSION

The CRISPR-Cas system is a typical immune system that can protect bacteria and archaea against invading foreign DNA. As an important element in the evolution process of prokaryotic organisms, how does a host distinguish between the advantages and disadvantages of a CRISPR-Cas system? Recently, the origin of diverse spacers and the mechanism of spacer acquisition have become the focus of attention. Bioinformatic analysis shows that in addition to attacking conjugative plasmid and bacteriophage, a small number of spacers match with archaeal or bacterial genomes (18, 19, 38, 39). Remarkably, although only a minority of spacers share homology with prokaryotic genomes, they present at a high frequency. About one in every 5.5 CRISPR-positive organisms contains at least one spacer matching with archaeal or its own bacterial genome (18). However, a reasonable and convincing explanation for the existence of chromosome-targeting spacers has not been provided yet. One theory is that chromosomal targeting is detrimental and bacteria escape from autoimmunity at a severe fitness cost of CRISPR-Cas system inactivation (18). A few studies have provided experimental evidence to support this hypothesis. In *P. carbinolicus* type I-E CRISPR-Cas system, the CRISPR locus contains a spacer against the housekeeping gene *hisS*. Transformation of the artificial plasmid with spacers targeting *hisS* into a *Geobacter sulfurreducens* strain could inhibit its growth (21). Introduction of an artificial mini-CRISPR locus with a spacer against the beta-galactosidase gene in *S. solfataricus* by transfection caused growth inhibition, and the host cells can survive by eliminating the corresponding CRISPR locus (40). In addition, spacers against integrated MGEs exhibited unexpected effects. Although the type I-F CRISPR-positive *P. atrosepticum* contained a spacer completely complementary to an endogenous gene within genomic island HAI2, CRISPR lethality was abolished due to a single nucleotide mutation in the PAM. Engineering a CRISPR locus with a correct PAM could recover the deleterious effect and promote bacterial genome evolution (20). A similar result was observed in the *S. thermophilus* type II-A system. When an artificial spacer targeting *lacZ* located in the integrated genomic island was introduced, most of the transformants were killed. Lac survivors showed large-scale genome deletion via IS-dependent recombination (29). However, in the type III-B system, chromosome-targeting spacers could be used as a tool to silence endogenous genes instead of killing cells due to the fact that the target is RNA, not DNA (41).

In this study, we have demonstrated that chromosomal targeting by the type III-A CRISPR-Cas system is significantly deleterious. Chromosomal targeting was achieved by transforming plasmids containing engineered CRISPR arrays with chromosome-targeting spacers. Importantly, the resistance gene *mecA* within SCC*mec* is chosen as the target. Neither the activity of a CRISPR-Cas system against integrated SCC*mec* nor its consequence for genome-scale evolution has been detected before. We have revealed that the most common fitness cost corresponding to chromosomal targeting is deletion of the target sequence. It seems that chromosomal targeting can provide a great selective pressure for bacterial genome evolution. Other types of negative fitness cost were also observed, such as loss-of-function mutations in *cas* genes and deletion of responsible spacers (Fig. 4D and E). Nevertheless, we did not observe any transposon insertion mutation or the deletion of the entire CRISPR-Cas locus among all 128 transformants. In a very small proportion of survivors, no mutation was found in protospacers, *cas* genes, or plasmids carrying a mini-CRISPR array. It seems reasonable to assume that CRISPR-Cas immunity is not absolutely abolished in these strains and that partial immunity leads to tolerance of self-targeting, which is in agreement with the results reported in *S. epidermidis* (22). Also, the proportion of different types of mutations in our experiments (Fig. 4A) differed from those observed by others in *S. epidermidis* and in *Sulfolobus islandicus* (22, 42). These results suggest that bacteria deal with the evolution downside of selective pressure through different mechanisms and produce preference according to differential conditions (targeting conjugative plasmid or chromosome). Moreover, bacteria can escape from chromosomal targeting at the negative cost of loss-of-function mutations in diverse *cas* genes. In addition, multiple point mutations were identified within the *cas1* gene (Table 2), which is not responsible for CRISPR immunity.

Among staphylococcal strains with type III-A CRISPR-Cas systems, most strains contain two CRISPR arrays with 14 or 15 spacers upstream and downstream of the *cas* locus, respectively (17). However, *S. aureus* strain AH1 has only one CRISPR array with three spacers. Similarly, *S. epidermidis* strain RP62A has only five spacers, three spacers located upstream of the *cas* locus and two spacers located downstream of *cas* (35). The number of CRISPR arrays and spacers may be associated with the background, environment, and evolution process of different strains. However, it does not influence the immunity function of the CRISPR-Cas system in different strains (17, 36). The sizes of the three native spacers were 35 or 37 nt in *S. aureus* strain AH1 (17). By changing the length of chromosome-targeting spacers in our experiments, we found that it had no influence on the size of mature crRNAs. Northern blot results showed two clear bands with sizes of about 43 and 37 nt as previously described (11). RACE assays further confirmed Northern blot results, indicating that the sizes of mature crRNAs are constant. We further demonstrated that spacer length has an effect on the targeting activity. The artificial spacers with the sizes of 36 or 33 nt exhibited high targeting capacity and triggered more than 99% of DNA degradation (Fig. 6D). Introduction of 13-nt mismatches between the target gene and the 3′ ends of crRNAs by truncating the spacer length to 22 nt could still result in more than 75% of DNA degradation (Fig. 6D). Further truncation (17 to 21 nt) completely abrogated CRISPR attack (Fig. 6D), indicating that more than 13 consecutive mutations in the 3′ ends of crRNAs can fully abolish CRISPR targeting activity. Similar conclusions were proposed in previous studies. For example, Cao et al. demonstrated that 12 consecutive nucleotide mutations resulted in a decreased immunity activity in *S. aureus* and that 13 consecutive nucleotide mutations completely disrupted CRISPR antiplasmid immunity (17). Manica et al. reported that more than 15 nucleotide mutations fully blocked CRISPR interference in *S. solfataricus* (16). These observations imply that mutations are highly tolerated between crRNAs and their protospacers and that the number of paired nucleotides between the crRNAs and protospacers is the decisive characteristic for CRISPR targeting.

The CRISPR-Cas system is a simple but ingenious defense system, and it can precisely discriminate self/nonself to prevent autoimmunity. In type I and II systems, host distinguishes self from nonself via the recognition of specific nucleotides in the PAM region. The type III CRISPR-Cas system is independent of the PAM and identifies targets by a distinctive mechanism. In *S. epidermidis*, three or more successive base parings between the 5′ tags of crRNAs and targets are necessary for self-recognition (15). One previous study has indicated that base pairing at positions −2, −3, and −4 is crucial and that this recognition process is independent of the nucleotide sequence (15). In *S. solfataricus*, similar conclusions are proposed but for positions −3, −4, and −5 (16). To figure out the key nucleotides for self/nonself discrimination in our strain, we constructed a chromosome-targeting spacer with multiple mutations in the first 8 nt of the repeat and performed the transformation experiments. Significantly higher transformation efficiencies were observed, suggesting that any consecutive three-nucleotide complementarity between the 5′ tag of crRNAs and the adjacent region of protospacers can block attack (Fig. 7B). This self-recognition was independent of position or sequence (Fig. 7). Interestingly, most of these transformants exhibited small and rough colonies, and further experiments confirmed that only ~50% chromosome degradation was realized in these clones, implying that CRISPR attack was not completely abolished (Fig. 7C). These data imply that the mechanism of self/nonself recognition in the type III CRISPR-Cas system is more complicated than we thought.

In conclusion, we use engineered chromosomal targeting as an alternative strategy to investigate the immunity function and molecular mechanisms of the type III-A CRISPR-Cas system in *S. aureus*. Our findings indicate that chromosomal targeting can drive large-scale deletion within integrated SCC*mec* and contribute to bacterial genome reshaping. In addition, this study may provide a promising tool to delete resistance and virulence genes in bacterial pathogens by CRISPR-Cas systems.

## MATERIALS AND METHODS

### Bacterial strains, plasmids, and growth conditions.

The bacterial strains and plasmids used in this study are listed in Table 4. *Escherichia coli* was grown (220 rpm) in lysogeny broth medium (Franklin Lakes) or on lysogeny broth agar (LA) at 37°C. *Staphylococcus aureus* strains were grown (220 rpm) in tryptic soy broth (TSB) (Difco) or on tryptic soy agar plates (Difco) at 37°C. When needed, 150 μg/ml ampicillin sodium salt or 50 μg/ml kanamycin sulfate for *E. coli* or 15 μg/ml chloromycetin for *S. aureus* strains was added to the bacterial cultures.

**TABLE 4:** Bacterial strains and plasmids used in this study

Strain or plasmid(s)	Characteristics^a^	Source or reference^b^
Strains		
*E. coli Trans*T1	Clone host strain; F^−^ φ80(*lacZ*) ΔM15 Δ*lacX74 hsdR* (r_K_^−^ m_K_^+^) Δ*recA1398 endA1 tonA *	TransGen
*S. aureus*		
RN4220	8325-4; restriction-negative strain	NARSA
AH1	CA-MRSA; SCC*mec* type V	Hospital
Δ*cas6*	AH1; *cas6*-deleted strain	
Plasmids		
pLI50	Shuttle vector; Amp^r^ Chl^r^	46
pLIC-404	pLI50 derivative with 404 bp of leader sequence and native CRISPR locus from *S. aureus* strain AH1	This study
pLIC-252	pLI50 derivative with 252 bp of leader sequence and native CRISPR locus from *S. aureus* strain AH1	This study
pLIC-158	pLI50 derivative with 158 bp of leader sequence and a native CRISPR array from *S. aureus* strain AH1	This study
pLI-252	pLI50 derivative with 252 bp of leader sequence and an artificial CRISPR array targeting *mecA*	This study
pLI-C	pLI50 derivative with an artificial CRISPR array targeting the coding strand of *mecA*	This study
pLI-T	pLI50 derivative with an artificial CRISPR array targeting the template strand of *mecA*	This study
pLI-1	pLI50 derivative with an artificial CRISPR array containing one spacer targeting *mecA*	This study
pLI-11	pLI50 derivative with artificial CRISPR arrays containing two identical spacers targeting *mecA*	This study
pLI-12	pLI50 derivative with artificial CRISPR arrays containing two different spacers targeting *mecA*	This study
pLI-S17, pLI-S20, pLI-S21,pLI-S22, pLI-S23, pLI-S25, pLI-S33, pLI-S36, pLI-S39, pLI-S42, pLI-S45	LI50 derivative containing *mecA*-targeting spacers with the spacer length of 17, 20, 21, 22, 23, 25, 33, 36, 39, 42, or 45 nt	This study
pLI-M1, pLI-M2, pLI-M3, pLI-M4, pLI-M5, pLI-M6, pLI-M7	pLI-S36 derivative with different mutations in the first repeat sequence	This study
pEASY blunt simple	Commercial cloning vector; Amp^r^ Kan^r^	TransGen

a CA-MRSA, community-associated MRSA; Amp^r^, ampicillin resistant; Chl^r^, chloramphenicol resistant; Kan^r^, kanamycin resistant.

b NARSA, Network on Antimicrobial Resistance in *Staphylococcus aureus*.

### Construction of artificial CRISPR arrays.

To construct CRISPR plasmids that can be used further for cloning and expression of any spacer and repeat sequence, 404, 252, or 158 bp of the native CRISPR leader and CRISPR arrays were amplified with forward primers leader404-f (f stands for forward), leader252-f, or leader158-f and the reverse primer CRISPR-r (r stands for reverse). The products were then digested with KpnI and SacI and ligated to pLI50 previously digested with the same enzymes, generating plasmids pLIC-404, pLIC-252, and pLIC-158. These plasmids were then digested with ClaI and ligated with engineered spacer repeat units, yielding artificial CRISPR plasmids pLI-252 and pLI-158. The repeat and target-specific spacer regions were amplified by PCR with the primer pairs that contained engineered spacer repeat units. The repeats were digested with the enzyme Cla, which resulted in the introduction of subsequent spacer repeat units, and this procedure could be performed to construct any artificial CRISPR array. These plasmids were first introduced into *S. aureus* strain RN4220 for modification and subsequently transformed into *S. aureus* strain AH1 and its mutant strains. All plasmids extracted from *S. aureus* strain RN4220 were sequenced to confirm that no mutation occurred during the modification process. The sequences of the primers used in plasmid construction are shown in Table 5.

**TABLE 5:** Primers used in this study

Primer	Sequence (5′–3′)^a^	Application
Leader404-f	CGGggtaccCATCTCAATTAAGCAGCTA	Amplification for 404-bp leader
Leader252-f	CGGggtaccCACCTAACTCACTATCAAT	Amplification for 252-bp leader
Leader158-f	CGGggtaccCGTATTAAATGTAGTATACT	Amplification for 158-bp leader
CRISPR-r	CCGgagctcCCATCCCCTAAAAATTAATCC	Amplification for a native CRISPR array
CRISPR-Cas-f1	TAACTCACTATCAATCATTTCTCCAC	Amplification for CRISPR-Cas locus
CRISPR-Cas-r1	GCATAATCCATCATCATTAATATCTATG	Amplification for CRISPR-Cas locus
CRISPR-Cas-f2	TATAGAACTATTTGGCGTAATG	Amplification for CRISPR-Cas locus
CRISPR-Cas-r2	GTAATCTTGCTTCTTCATAACT	Amplification for CRISPR-Cas locus
CRISPR-Cas-f3	TTTATGGTTGGAGGTATAAGTATGAC	Amplification for CRISPR-Cas locus
CRISPR-Cas-r3	TATATTATACTATATTTCCCCATGCC	Amplification for CRISPR-Cas locus
R1-S1-f	GATCGATAACTACCCCGAAGAATAGGGGACGAGAACAATCAGTATTTCACCTTGTCCGTAACCTGAATCAG	pLI-1, pLI-11, pLI-12
S1-R2-r	CACTCTGTCCCCTATTCTTCGGGGTAGTTATCGATCCTGATTCAGGTTACGGACAAGGTGAAATACTGATT	pLI-1,pLI-11, pLI-12
R2-S2-f	GATCGATAACTACCCCGAAGAATAGGGGACGAGAACCAATATGTATGCTTTGGTCTTTCTGCATTCCTGGA	pLI-12
S2-R2-r	CACTCTGTCCCCTATTCTTCGGGGTAGTTATCGATCTCCAGGAATGCAGAAAGACCAAAGCATACATATTG	pLI-12
R1-*mecA*C-f	GATCGATAACTACCCCGAAGAATAGGGGACGAGAACGCAGTACCGGATTTGCCAATTAAGTTTGCATAA	pLI-C
*mecA*C-R2-r	CACTCTGTCCCCTATTCTTCGGGGTAGTTATCGATCTTATGCAAACTTAATTGGCAAATCCGGTACTGC	pLI-C
R1-*mecA*T-f	GATCGATAACTACCCCGAAGAATAGGGGACGAGAACTTATGCAAACTTAATTGGCAAATCCGGTACTGC	pLI-T
*mecA*T-R2-r	CACTCTGTCCCCTATTCTTCGGGGTAGTTATCGATCGCAGTACCGGATTTGCCAATTAAGTTTGCATAA	pLI-T
R1-S17-f	GATCGATAACTACCCCGAAGAATAGGGGACGAGAACATTTCACCTTGTCCGTA	pLI-S17
S17-R2-r	CACTCTGTCCCCTATTCTTCGGGGTAGTTATCGATCTACGGACAAGGTGAAAT	pLI-S17
R1-S20-f	GATCGATAACTACCCCGAAGAATAGGGGACGAGAACATTTCACCTTGTCCGTAACC	pLI-S20
S20-R2-r	CACTCTGTCCCCTATTCTTCGGGGTAGTTATCGATCGGTTACGGACAAGGTGAAAT	pLI-S20
R1-S21-f	GATCGATAACTACCCCGAAGAATAGGGGACGAGAACATTTCACCTTGTCCGTAACCT	pLI-S21
S21-R2-r	CACTCTGTCCCCTATTCTTCGGGGTAGTTATCGATCAGGTTACGGACAAGGTGAAAT	pLI-S21
R1-S22-f	GATCGATAACTACCCCGAAGAATAGGGGACGAGAACATTTCACCTTGTCCGTAACCTG	pLI-S22
S22-R2-r	CACTCTGTCCCCTATTCTTCGGGGTAGTTATCGATCCAGGTTACGGACAAGGTGAAAT	pLI-S22
R1-S23-f	GATCGATAACTACCCCGAAGAATAGGGGACGAGAACATTTCACCTTGTCCGTAACCTGA	pLI-S23
S23-R2-r	CACTCTGTCCCCTATTCTTCGGGGTAGTTATCGATCTCAGGTTACGGACAAGGTGAAAT	pLI-S23
R1-S25-f	GATCGATAACTACCCCGAAGAATAGGGGACGAGAACATTTCACCTTGTCCGTAACCTGAAT	pLI-S25
S25-R2-r	CACTCTGTCCCCTATTCTTCGGGGTAGTTATCGATCATTCAGGTTACGGACAAGGTGAAAT	pLI-S25
R1-S33-f	GATCGATAACTACCCCGAAGAATAGGGGACGAGAACTAATCAGTATTTCACCTTGTCCGTAACCTGAAT	pLI-S33
S33-R2-r	CACTCTGTCCCCTATTCTTCGGGGTAGTTATCGATCATTCAGGTTACGGACAAGGTGAAATACTGATTA	pLI-S33
R1-S36-f	GATCGATAACTACCCCGAAGAATAGGGGACGAGAACTAATCAGTATTTCACCTTGTCCGTAACCTGAATCAG	pLI-S36
S36-R2-r	CACTCTGTCCCCTATTCTTCGGGGTAGTTATCGATCCTGATTCAGGTTACGGACAAGGTGAAATACTGATTA	pLI-S36
R1-S39-f	GATCGATAACTACCCCGAAGAATAGGGGACGAGAACTAATCAGTATTTCACCTTGTCCGTAACCTGAATCAGCTA	pLI-S39
S39-R2-r	CACTCTGTCCCCTATTCTTCGGGGTAGTTATCGATCTAGCTGATTCAGGTTACGGACAAGGTGAAATACTGATTA	pLI-S39
R1-S42-f	GATCGATAACTACCCCGAAGAATAGGGGACGAGAACTAATCAGTATTTCACCTTGTCCGTAACCTGAATCAGCTAATA	pLI-S42
S42-R2-r	CACTCTGTCCCCTATTCTTCGGGGTAGTTATCGATCTATTAGCTGATTCAGGTTACGGACAAGGTGAAATACTGATTA	pLI-S42
R1-S45-f	GATCGATAACTACCCCGAAGAATAGGGGACGAGAACTAATCAGTATTTCACCTTGTCCGTAACCTGAATCAGCTAATAATA	pLI-S45
S45-R2-r	CACTCTGTCCCCTATTCTTCGGGGTAGTTATCGATCTATTATTAGCTGATTCAGGTTACGGACAAGGTGAAATACTGATTA	pLI-S45
R1-S36m1-f	GATCGATAACTACCCCGAAGAATAGGGGACGACAACTAATCAGTATTTCACCTTGTCCGTAACCTGAATCAG	pLI-M1
R1-S36m2-f	GATCGATAACTACCCCGAAGAATAGGGGACGAGGACTAATCAGTATTTCACCTTGTCCGTAACCTGAATCAG	pLI-M2
R1-S36m3-f	GATCGATAACTACCCCGAAGAATAGGGGACGAGGGCTAATCAGTATTTCACCTTGTCCGTAACCTGAATCAG	pLI-M3
R1-S36m4-f	GATCGATAACTACCCCGAAGAATAGGGGACGAGTTCTAATCAGTATTTCACCTTGTCCGTAACCTGAATCAG	pLI-M4
R1-S36m5-f	GATCGATAACTACCCCGAAGAATAGGGGACGACGGTTAATCAGTATTTCACCTTGTCCGTAACCTGAATCAG	pLI-M5
R1-S36m6-f	GATCGATAACTACCCCGAAGAATAGGGGACGTGGACTAATCAGTATTTCACCTTGTCCGTAACCTGAATCAG	pLI-M6
R1-S36m7-f	GATCGATAACTACCCCGAAGAATAGGGGTACAGAACTAATCAGTATTTCACCTTGTCCGTAACCTGAATCAG	pLI-M7
*mecA*-f	TAATAGTTGTAGTTGTCGGGTTTGG	*mecA* detection
*mecA*-r	CATCGTTACGGATTGCTTCACTGTT	*mecA* detection
*cas6*-f	TTTAGGAAGTATTTTACATGGCGTG	*cas6* detection
*cas6*-r	CCAGAAAATTCACCAAACTTCAATA	*cas6* detection
CRISPR-RT-f	GGGACGAGAACTTCAAAT	qRT-PCR
CRISPR-RT-r	CAGTATGAAACAAATCAAGGT	qRT-PCR
*mecA*-r-biotin	ATTCAGGTTACGGACAAGGTGAAATACTGATTA	Northern blotting

a Nucleotides in the restriction sites are indicated by lowercase letters.

### Preparation of electrocompetent *S. aureus* cells.

*S. aureus* cells from 15% glycerol stock were streaked on a TSB agar plate and incubated at 37°C. A single colony was selected and incubated in 5 ml TSB at 37°C overnight. One-milliliter portions of the overnight culture were added to 100 ml TSB in a 500-ml flask and shaken at 37°C until an optical density at 600 nm (OD_600_) of 0.4 was reached. The culture was put on ice for 5 min and then transferred to a sterile, round-bottom centrifuge tube. The cells were collected by centrifugation at 2,500 ×  *g* at 4°C for 10 min, and the supernatant was discarded. The cells were gently resuspended in 10 ml of ice-cold 0.5 M sucrose, and the suspension was kept on ice for 5 min. The centrifugation and resuspension steps were repeated twice. The cells were then resuspended in 1 ml of ice-cold 0.5 M sucrose, and the suspension was kept on ice for 15 min. Finally, 100-μl aliquots were prepared in sterile microcentrifuge tubes and frozen in liquid nitrogen. The competent cells were stored at −80°C.

### Plasmid extraction and transformation in *S. aureus.*

Plasmids from all *S. aureus* strains were isolated using a plasmid purification kit (Sangon Biotech) according to the manufacturer’s instructions, except that the cells were pretreated with digestion buffer containing 40 U/ml lysostaphin, 10 mg/ml lysozyme, and 10% (vol/vol) glycerol for 30 to 60 min. Plasmids were transformed into all *S. aureus* strains by electroporation. Plasmid DNA (100 to 500 ng) and electrocompetent *S. aureus* cells (100 μl) were mixed and placed in a Gene Pulser cuvette with a 0.2-cm electrode gap. The settings for electroporation are as follows: voltage, 2.5 kV; capacitor, 50 μF; resistance, 200 Ω. After electroporation, 400 μl TSB was immediately added to the cuvette, and the cuvette was put on ice for 15 min. The cells were then transferred into a 1.5-ml Eppendorf tube and incubated with shaking (220 rpm, 37°C) for 1 h before being spread on a TSB plate.

### Oxacillin susceptibility assay.

The oxacillin susceptibility of the WT strain and transformants was evaluated by detecting the microbroth MIC of oxacillin according to Clinical and Laboratory Standards Institute (CLSI) criteria (43). The cultures of all strains were diluted to a final test concentration of approximately 5 × 10^4^ CFU/well and incubated at 37°C for 24 h.

### Total RNA extraction and qRT-PCR.

Total RNA was extracted by RNAiso plus according to the manufacturer’s instructions (TaKaRa). Residual DNA was digested with RNase-free DNase I (TaKaRa). Reverse transcription was carried out with the PrimeScript first-strand cDNA synthesis kit (TaKaRa), and real-time PCR was performed with SYBR Premix *Ex Taq* (TaKaRa) using a StepOne real-time system (Applied Biosystems). The quantity of cDNA was normalized to the abundance of *pta* cDNA (44). All the qRT-PCR assays were repeated at least three times.

### Evaluation of DNA targeting efficiency by real-time PCR.

To analyze the ratio of *mecA*-positive clones in the *S. aureus* population, strains carrying *mecA*-targeting constructs were cultivated in TSB with chloromycetin (15 μg/ml) at 37°C for 24 h, then cells were collected, and genomic DNA was extracted. A final concentration of 200 ng/ml genomic DNA was used as the template. The real-time PCR was performed with SYBR Premix *Ex Taq* (TaKaRa) using the StepOne real-time PCR system (Applied Biosystems). The quantity of *mecA* measured by real-time PCR was normalized to the abundance of *pta* DNA (44). All the real-time PCR assays were repeated at least three times. The relative targeting activity of *mecA*-targeting spacer was equal to one minus the value of the relative quantity of *mecA*.

### Northern blot analysis.

Total RNA (30 mg) was denatured at 95°C for 5 min and then separated with a 12% denatured polyacrylamide–7 M urea gel (100 V, 1.5 h) in 1× Tris-borate-EDTA (TBE) and transferred onto a nylon membrane in 0.5× TBE. The product was then immobilized by UV cross-linking and blotted with the biotin-labeled oligonucleotide probes. RNA-DNA hybridization detection using a North2South chemiluminescence hybridization and detection kit (Thermo Scientific) was performed to detect crRNAs.

### Determination of mature crRNA sequences by RACE.

The 5′ and 3′ ends of crRNAs were determined by RACE using the full 3′ RACE core set version 2.0 and the full 5′ RACE kit (TaKaRa) as previously described (45). PrimeSTAR HS DNA polymerase (TaKaRa) was used for PCR amplification, and the amplified RACE products were ligated with pEASY-Blunt Simple Cloning vector (pEASY-Blunt Simple Cloning kit; TransGen Biotech). The ligation was transformed into *E. coli Trans*T1, and transformants were characterized by colony PCR to amplify the RACE products. The positive colonies were sequenced using the M13 forward sequencing primer (Sangon Biotech).

### Genomic DNA extraction and sequencing.

Genomic DNA was extracted and sequenced using an Illumina Hiseq 2000 platform (Institute of Microbiology, Chinese Academy of Sciences, Beijing, China). About 1.8 GB of high-quality sequence data of each genome was then mapped using SOAP (short oligonucleotide alignment program; BGI) software.

### Statistical analysis.

First, F test for two samples was performed for variances. Unpaired two-tailed *t* test for equal or unequal variances was then performed to calculate the significant differences (*P* value). All the tests were performed by the data analysis tool in Microsoft Excel.
